# Learning Macromanagement in Starcraft by Deep Reinforcement Learning

**DOI:** 10.3390/s21103332

**Published:** 2021-05-11

**Authors:** Wenzhen Huang, Qiyue Yin, Junge Zhang, Kaiqi Huang

**Affiliations:** 1 School of Artificial Intelligence, University of Chinese Academy of Sciences, Beijing 100049, China; huangwenzhen2014@ia.ac.cn (W.H.); qyyin@nlpr.ia.ac.cn (Q.Y.); jgzhang@nlpr.ia.ac.cn (J.Z.); 2 CRISE, Institute of Automation, Chinese Academy of Sciences, Beijing 100190, China; 3 CAS Center for Excellence in Brain Science and Intelligence Technology, Beijing 100190, China

**Keywords:** StarCraft, macromanagement, Asynchronous Advantage Actor-Critic

## Abstract

StarCraft is a real-time strategy game that provides a complex environment for AI research. Macromanagement, i.e., selecting appropriate units to build depending on the current state, is one of the most important problems in this game. To reduce the requirements for expert knowledge and enhance the coordination of the systematic bot, we select reinforcement learning (RL) to tackle the problem of macromanagement. We propose a novel deep RL method, Mean Asynchronous Advantage Actor-Critic (MA3C), which computes the approximate expected policy gradient instead of the gradient of sampled action to reduce the variance of the gradient, and encode the history queue with recurrent neural network to tackle the problem of imperfect information. The experimental results show that MA3C achieves a very high rate of winning, approximately 90%, against the weaker opponents and it improves the win rate about 30% against the stronger opponents. We also propose a novel method to visualize and interpret the policy learned by MA3C. Combined with the visualized results and the snapshots of games, we find that the learned macromanagement not only adapts to the game rules and the policy of the opponent bot, but also cooperates well with the other modules of MA3C-Bot.

## 1. Introduction

**Figure 1:**
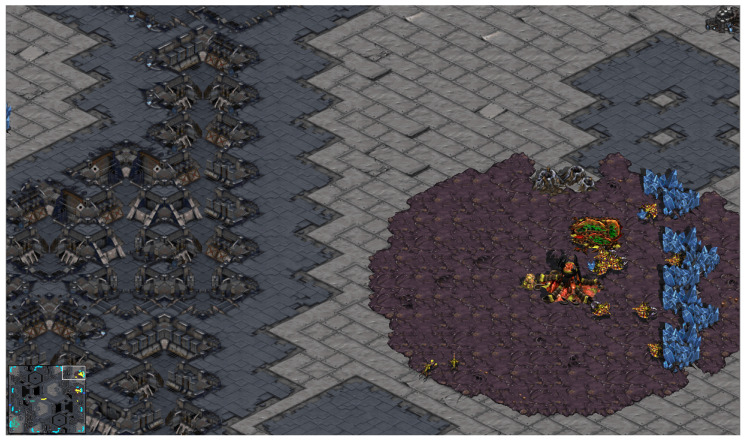
A screenshot of StarCraft.

StarCraft is a Real-Time Strategy (RTS) game that was released by Blizzard Entertainment in 1998. Figure 1 shows a screenshot of StarCraft. Similar to other RTS games, the core tasks in StarCraft are gathering resources, training the military, and using them to defeat the opponent’s army. When compared with traditional board games or Atari games, StarCraft has a larger state space and more high-dimensional control interfaces. In addition, the states of this game are partially observable. Although AlphaStar [1] can reach the human level in the game of StarCraft II, its success is extremely dependent on huge computing resources. It is worth studying how to decompose the complex problem of the StarCraft game into several sub-problems, so as to solve it more efficiently [2,3,4].

Macromanagement is one of the most important sub-problems. Macromanagement aims at deciding what to build or which combat units to produce, depending on the current state including buildings and units of both sides, the resources and the technologies of our side. In StarCraft, each unit has its own strengths and weaknesses, while each type of building has its own unique function. Making the appropriate selection based on the current state is the foundation of victory. Thus, we focus on macromanagement tasks.

Using hand-coded strategies is a common method of macromanagement, and it is widely applied in the StarCraft bots coding in AI competitions [2,5]. Obviously, this method needs the designers’ professional knowledge and a large amount of time. With the development of computational intelligence in recent years, machine learning [6], deep learning [7,8], and reinforcement learning [9,10] have been introduced into the field of StarCraft AI design [11]. Tree search is applied for optimizing build orders to reach a specific goal [12]. The replay data of the professional players provide valuable training samples, thus Supervised Learning (SL) methods can learn macromanagement decisions from these data [13]. SL methods can obtain the decision models without many hand-crafted rules, but they also rely on labeled data. It should be noted that human players’ decision is based on their ability to solve other sub-problems, such as micromanagement and resource management. For the AI bots, the capability of the modules that are designed to solve these sub-problems are poorer than humans. For instance, professional players prefer strong but complex army units, while most of the bots are unable to control these units effectively. Thus, the choices made by human players may be inappropriate for the other modules. Evolutionary Computation (EC) can produce the optimal solutions under a wide range of problem settings and it does not need labeled data. Some methods use the win rate or the in-game scores as the fitness metrics and apply EC to evolve the complete building plan [14,15]. Reinforcement Learning (RL) methods are designed to solve sequential decision problems and they are more sample-efficient than EC methods, so RL methods are very suitable for solving the macromanagement task [16]. We select RL to learn macromanagement in order to reduce the dependence on professional knowledge and enhance the adaptability to other modules.

Reinforcement learning (RL) is a powerful tool for solving sequential decision-making problems and it has been widely used in various fields. The recent research work on the combination of reinforcement learning and deep learning has made tremendous achievements. Mnih et al.  [17] proposed Deep Q-Network (DQN) and trained an agent that performs comparably to human players on Atari games. AlphaGo [18] won the Go competition to a world champion for the first time. In addition, an extraordinary amount of novel algorithms [10,19,20,21,22,23,24,25] are proposed for improving the stability and performance of deep reinforcement learning algorithms, reduce training time, and expand the scope of application. In the context of StarCraft micromanagement, researchers have proposed a series of deep reinforcement learning methods and they have achieved the desired results [26,27,28].

For the problem of macromanagement, the rewards of victory or defeat can be obtained without any expert knowledge. The environment can be considered to be composed of the game and the bots of two sides. To obtain as many rewards as possible, the RL agent would choose the units to build that not only can restrict the opponent’s army, but can also be controlled by the micromanagement module. In this way, the learned macromanagement is adapted to not only the external environment, the game, and the opponent bot, but also the ability of the other modules in the bot.

However, there are still some technical difficulties in practice. Firstly, the agent can only obtain partial information of the full game state due to the ‘fog-of-war’. Secondly, the environment is stochastic for the agent to a certain extent, due to the problem of partial information and the uncertainty of other modules. Even the same states and actions may lead to different results. Finally, the training time is very long on account of that each match would take a relatively long time, approximately 3–10 min.

Essentially, the first issue is Partially Observable Markov Decision Processes (POMDPs) problem, which means that the states of the environment are only partially observable. Hausknecht and Stone [21] attempt to solve it through extracting state feature from the observation queue. However, the action queue of our agent also influences the actual game state. Thus, we use LSTM [29,30] to extract the feature of history queue combined with all observations and actions. The extracted feature can improve the precision of evaluation on the true state. Being inspired by [25], we update the policy utilizing the advantage function Q(s,a)−V(s) instead of the TD-error signal $V\left(s^{\prime }\right)+r\left(s,a\right)-V\left(s\right)$. The former one can be considered as the expectation of the latter one. Finally, we train multiple agents in parallel to shorten the training time and update the parameters of these agents using asynchronous gradient descent [24].

The main contributions of this paper are: (i) we introduce the RL method to solve the problem of macromanagement; (ii) we propose a novel deep RL method, Mean Asynchronous Advantage Actor-Critic (MA3C), which can solve the problem of imperfect information, the uncertainty of state transition, and the matter of long training time; and, (iii) we present an approach to visualize the policy learned by deep RL method.

## 2. Related Works

There are many challenges in designing AI bots for StarCraft, such as resource management, decision-making under uncertainty, adversarial real-time planning, and so on. Alphastar [1] uses massive computing resources to solve the problem of StarCraft as a whole to a certain extent, but it is also concerned to decompose the problem into multiple subproblems to solve it more efficiently. Many research works decompose designing AI bots into a series of subtasks: strategy, tactics, reactive control, terrain analysis, and intelligence gathering [2]. This paper focuses on macromanagement, which is one of the core problems in the ‘strategy’ subtask.

Hand-coding is the earliest solution of macromanagement and it has been widely applied in AI competitions [2,5]. These methods require considerable professional knowledge and need generous time for coding and debugging. To reduce the dependence on this knowledge, ref. [13] directly learns how macromanagement decisions from game replays using Supervised Learning (SL). When the learned network is integrated into a StarCraft bot, the system outperforms the game’s built-in bot. However, the macromanagement of human players is highly related to their unique micromanagement, resource management, etc. Thus, the network that is learned by SL may be inappropriate for the other modules of the bot that it integrated. Evolutionary Computation (EC) and Reinforcement Learning (RL) can avoid this problem. Ref. [14] presents a framework to evolve a complete strategy for StarCraft from the building plan to the composition of squads. Ref. [15] proposes an online evolutionary planning method to enable the algorithm to adapt to new situations. RL is more sample efficient when compared with EC. Ref. [16] utilizes the RL method, Double Q-Learning [23], to obtain some rational build-order arrangements according to the real-time situation in the game and fight against the built-in AI in simple maps. Ref. [1] proposes AlphaStar, which trains the policy network with imitation learning and then uses league style reinforcement learning to optimize the network further. AlphaStar is rated above 99.8% of officially ranked human players. However, AlphaStar is required to train on hundreds of TPUs for months. In order to reduce the computation needed, ref. [31] optimizes the network architecture and training methods, like reducing the input minimap size and adding the agent branching approach. Ref. [32] also proposes new league training methods and lightweight neural network architecture to train a competitive AI agent with limited computation resources.

Our method is most similar to [16]. The main goals of both [16] and our method are using RL to learn which unit should be built out. The main difference is the RL algorithms and the networks [16] selects Double Q-Learning to their network, while we propose a novel RL algorithm, MA3C, which runs multiple RL processes in parallel to reduce the training time and computes the approximate expected gradient to improve the stability of the algorithm. Besides, we add LSTM unit to our network to tackle the POMDP problem.

We compare these works in Table 1 from the following aspects, the category of the algorithm, training time, the learned policy appropriated for other modules, the required computing resource, the magnitude of parameters, and the opponent selected in evaluation.

## 3. Background

### 3.1. Reinforcement Learning

Reinforcement learning (RL) is a powerful tool to solve sequential decision-making problems in which an agent interacts with the environment over a number of discrete time steps. At each discrete time step *t*, the agent receives a state $s_{t}$ from the environment, and it responds an action $a_{t}$ selected from the action space A. Subsequently, the environment provides a new state $s_{t+1}$ and a reward $r_{t+1}$ according to the state transition probability $P(s^{\prime }|s,a)$ and reward function R(s,a), respectively. The agent’s goal is maximizing the expected discounted return $E(R_{t})$ through finding an optimal policy $\pi (a|s_{t})$. The discounted return $R_{t}=\sum_{k=0}^{\infty }\gamma^{k}r_{t+k+1}$ is defined as the discounted sum of future rewards obtained by the agent. Additionally, γ is the discount factor balancing the importance of immediate and long term rewards.

General RL algorithms can be split into two categories: value-based methods and policy-based methods.

In value-based RL methods, the optimal policy is obtained indirectly through acquiring the optimal action value $Q^{*}\left(s,a\right)$. The action value $Q^{\pi }\left(s,a\right)=E\left(R_{t}|s_{t}=s,a\right)$ is defined as the expected discounted return for the agent selecting action *a* in state *s* and then following the policy π from the next time t+1. Additionally, the optimal action value $Q^{*}\left(s,a\right)$ is equal to $\max_{\pi }Q^{\pi }\left(s,a\right)$. $Q^{*}\left(s,a\right)$ is usually approximated by a function approximator Q(s,a|θ). The parameters θ in the approximator are learned by minimizing the loss

(1) $${\left(r_{t+1}+\gamma \max_{a^{\prime }}Q\left(s_{t+1},a^{\prime };\theta^{-}\right)-Q\left(s_{t},a_{t};\theta \right)\right)}^{2}.$$

In policy-based RL methods, the parameters θ of the optimal policy π(a|s;θ) is obtained directly by maximizing the expected discounted reward $E(R_{t})$. Williams [34] shows that ${▿}_{\theta }\log\pi \left(a_{t}|s_{t};\theta \right)\left(R_{t}-b_{t}\left(s_{t}\right)\right)$ is an unbiased estimate of ${▿}_{\theta }E\left[R_{t}\right]$. $b_{t}\left(s_{t}\right)$ is a baseline measuring the performance of current policy, and it is usually set to $V^{\pi }\left(s_{t}\right)=E\left[R_{t}|s_{t}\right]$ for reducing the variance estimate of the policy gradient. Additionally, in practice, $R_{t}$ is approximated by $r_{t}+\gamma V\left(s_{t+1}\right)$. Accordingly, the common policy gradient is as following,

(2) $${▿}_{\theta }\log\pi \left(a_{t}|s_{t};\theta \right)\left(r_{t}+\gamma V\left(s_{t+1}\right)-V\left(s_{t}\right)\right)$$

#### 3.1.1. Deep Recurrent Q-Network (DRQN)

When the states of the environment are partially observable to the agent, the sequential decision-making problems are called a partially observable Markov decision process (POMDP). In some RL problems, the agent cannot obtain the full information of the state $s_{t}$, and they have to select an action $a_{t}$ based on observations $o_{t}$, which is generated by the current state $s_{t}$ and the previous state $s_{t-1}$ and action $a_{t-1}$ according to a certain probability distribution $o∼Z(s_{t-1},a_{t-1},s_{t})$.

Under such circumstances, a reasonable solution is making a decision based on the historical observation queue $h_{t}=\left(o_{0},o_{1},\cdots ,o_{t}\right)$. Therefore, Hausknecht et al. [21] introduce DRQN, which combined a Long Short Term Memory (LSTM) [29] and a Deep Q-Network (DQN) [17]. LSTM can memorize the information of the historical queue efficiently. Based on the output of LSTM, DQN can make a more accurate evaluation of the current state.

#### 3.1.2. Asynchronous Advantage Actor-Critic (A3C)

Mnih et al. proposed asynchronously running multiple RL processes in parallel to replace experience replay. This method not only stabilizes the training process, but it also reduces the dependence on specialized hardware, like GPUs and the training time in practice.

## 4. Approach

**Figure 2:**
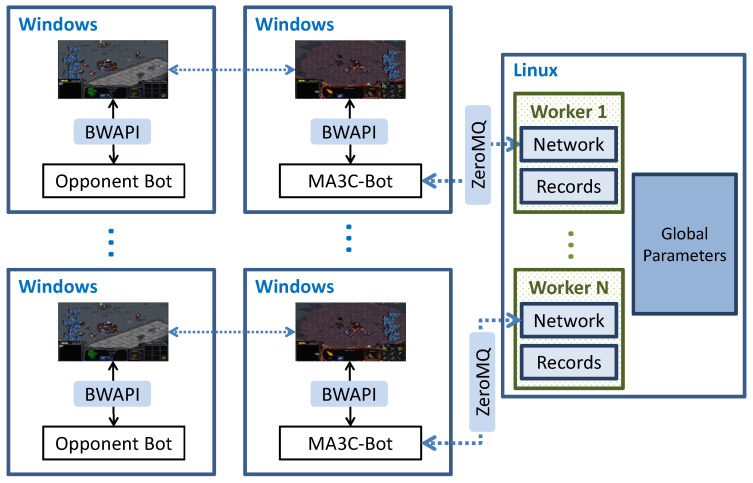
The architecture of the whole training system: the game platform, the opponent bot, and MA3C-bot run in the machines or virtual machines with Windows system. The algorithm of MA3C runs in the machine with Linux system. They communicate with each other by using Zeromq. MA3C contains multiple workers and a set of global shared network parameters. Each worker contains a local network and some records of recent experience data.

**Figure 3:**
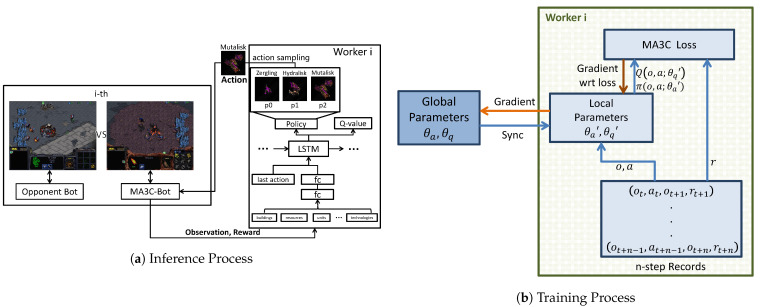
The inference and training processes of the algorithm of MA3C. **Inference Process**: when MA3C-Bot needs to decide which unit to produce, it sends the current observable game state and the reward to the RL agent in the according worker. The agent utilizes LSTM to encode the history queue—the queue of observations and actions. The encoded feature is used to calculate the policy—the probability of selecting different actions, and Q-value—the value of each action. An action is sampled according to the outputted policy, and then it is sent to MA3C-Bot and leads MA3C-Bot to produce the according unit. **Training Process**: every *n* actions sent, the recorded *n* tuples are used to compute the MA3C loss. The gradients of $\theta_{a}^{\prime }$, $\theta_{q}^{\prime }$ are computed and then sent to update the global parameters $\theta_{a}$, $\theta_{q}$.

Figure 2 shows the architecture of the whole training system. The game platform, the opponent bot, and MA3C-bot run in the machines or virtual machines with Windows system, where MA3C-Bot is a bot developed to interact with our RL agent. The algorithm of MA3C that was proposed by us runs in the machine with a Linux system. They communicate with each other by using ZeroMQ (https://zeromq.org/, accessed 10 April 2021). Figure 3 shows the inference and training processes of the algorithm, MA3C. Next, we will introduce this framework in details.

### 4.1. MA3C-Bot

MA3C-Bot uses a fixed opening strategy in the earlier period of a match. After finishing the opening build queue, the MA3C-Bot selects a kind of army unit every 24 frames and continuously produces this unit as long as the resources are enough. The selection is obtained by interacting with the RL agent. In detail, MA3C-Bot fetches the visible parts of the game state through BWAPI (https://github.com/bwapi/bwapi/tree/v4.1.2, accessed 10 April 2021), the API for interacting with Starcraft: Broodwar. Subsequently, it sends the observation to the RL agent and receives the decision from the agent by using ZeroMQ, a high-performance asynchronous messaging library. It is noted that MA3C-Bot and the agent are running separately, which is convenient for training multiple agents in parallel.

### 4.2. Rewards

The rewards are kept to be 0 except when the game ends. When the bot wins, the agent gets a positive reward, ‘+1’. And when the bot loses, the agent gets a negative reward, ‘−1’. To reduce the dependence on expert knowledge, we do not introduce other rewards.

### 4.3. Action Space

Although macromanagement means selecting which buildings or units to produce depending on the current state, the action space in this paper does not include any buildings. Because the MA3C-Bot is not able to efficiently use the complex army units, like Queens, and it does not need to produce the relevant buildings, like Queen’s Nest. Thus, the construction of buildings mainly occurs in the earlier period. For the selection of buildings in the earlier period, we directly select a fixed opening strategy. The is because seeking a novel and useful opening strategy by RL is difficult and time-consuming. There is an enormous amount of opening strategies explored by human players, so we can easily obtain an opening strategy that is appropriate to the MA3C-Bot.

The action space is reduced to three important army units: Zergling, Hydralisk, and Mutalisk. The reduction of the action space results in the reduction of the space of feasible policy, and so the algorithm can learn the appropriate policy more efficiently.

### 4.4. Observations

In this problem, the main factors affecting decision-making are the number of different types of buildings, units, and resources (mineral, gas, and supply), the level of different technologies, the races of both sides, the map size, and the current time.

We encode the “discrete” features, the tech levels, and the races using the binary code. Additionally, we encode the “continuous” features using the raw value, like the map size and the number of different types of buildings, units, and resources. For continuous features, we keep the moving averages and variances and normalize the features when the RL agent obtains them from MA3C-Bot. The discrete features and normalized continuous features are concatenated as the observations and are fed to the deep neural network.

All of the features that we selected are shown in Table 2. In the table, ‘Our*X*’ or ‘Enemy*X*’ means the number of the units/buildings ‘*X*’ in our or enemy’s side. Additionally, ‘has*X*’ means whether the buildings or technology, named ‘*X*’, exist on our side. In order to enhance the scalability of our algorithm, we select all the possible units, buildings, and technologies as the futures. Even so, the dimension of features is only about 580, which would not reduce the training efficiency.

### 4.5. Policy Network

The RL agent can obtain full information of our side, but it cannot obtain accurate information of the opponent’s side. To solve the POMDP problem, we utilize LSTM to extract feature of the history queue, $h_{t}\;=\;<a_{0},o_{1},a_{1},o_{2},\cdots ,a_{t-1},o_{t}>$, which is combined with all observations and actions from the beginning of a match to current time. The knowledge that is extracted from the history queue can improve the precision of evaluation on the real state. Take a simple example: a number of army units of the opponent are found a few seconds ago, but then they are invisible for ’fog-of-war’. The history queue could make the agent aware of the existence of them.

More concretely, the observation vector $o_{t}$ that is received from MA3C-Bot is fed into a two-layer fully-connected network. The output, a 512-D vector, is concatenated with the last action vector $a_{t-1}$ and fed into an LSTM layer. The action vector is encoded using the “one-hot” coding. Subsequently, the 512-D output of LSTM is fed into two fully-connected layers, respectively. One of them outputs Q-values for all possible actions, $Q(a_{t-1},o_{t},a;\theta_{q})$. For the other one, a softmax function is employed and the final output is the probabilities for each action, $\pi (a_{t}|a_{t-1},o_{t};\theta_{a})$. $\theta_{q}$ and $\theta_{a}$ share the weights of the two-layer fully-connected network and the LSTM layer. Additionally, they also contain the parameters of two output layers, respectively.

Although the network can only calculate the Q-value $Q(a_{t-1},o_{t},a)$ and the policy $\pi (a_{t-1},o_{t},a)$, the value of the current state can be calculated through:

(3) $$V\left(a_{t-1},o_{t}\right)=\sum_{a\in A}\pi \left(a_{t-1},o_{t},a\right)Q\left(a_{t-1},o_{t},a\right).$$

### 4.6. Mean Asynchronous Advantage Actor-Critic

Now, we introduce how to optimize the policy net by our proposed Mean Asynchronous Advantage Actor-Critic (MA3C). One match of StarCraft takes a relatively long time, about 3–10 min., thus training the policy net would take much time. To reduce the training time, we train *N* agents in parallel and update the parameters of the network using asynchronous gradient descent [24]. Each agent runs on a separate thread, interacts with a corresponding MA3C-Bot, and has its own copy of the policy net. The parameters of the policy net are denoted as $\theta_{a}^{\prime }$, $\theta_{q}^{\prime }$. The main process saves the global shared parameters $\theta_{a}$, $\theta_{q}$. The gradients calculated in all threads would be used to update the shared parameters $\theta_{a}$, $\theta_{q}$.

MA3C uses the n-step returns to update the policy network. For computing a single update, MA3C first copies the parameters of the network from the main process and then selects actions for *n* steps. The n-step Q-loss is

(4) $$\begin{array}{cc} L_{nstep-Q}=\sum_{i=1}^{n} & \left(Q(a_{t+n-i-1},o_{t+n-i},a_{t+n-i};\theta_{q}^{\prime }\right)\\ \; & -\sum_{j=1}^{j}\left[\gamma^{i-j}r_{t+n-j}\right]-\gamma^{i}V\left(a_{t+n-1},o_{t+n};\theta_{a}^{\prime },\theta_{q}^{\prime }\right){)}^{2}. \end{array}$$

The term $V(a_{t+n-1},o_{t+n};\theta_{a}^{\prime },\theta_{q}^{\prime })$ would be used as a constant value when the algorithm takes the derivative of this loss with respect to $\theta_{q}^{\prime }$.

For the same current observation $o_{t}$ and action $a_{t}$, the next observation $o_{t+1}$ is still nondeterministic. The history queue is short in the early stage of a match, so LSTM only reduces uncertainty to a certain extent. To improve the stability of our algorithm, we replace the TD-error signal $V\left(s^{\prime }\right)+r\left(s,a\right)-V\left(s\right)$ in the Actor–Critic method with its approximate expectation on the distribution of $p(s^{\prime }|s,a)$ like [25]. We also add the entropy of the policy to avoid it converging to a sub-optimal deterministic one. The policy gradient is

$$\begin{array}{cc} \Delta \theta_{a}^{\prime }=\sum_{i=1}^{n}{▿}_{\theta_{a}^{\prime }}\{ & \sum_{a\in A}\left[Q\left(a_{t+i-1},o_{t+i},a;\theta_{q}^{\prime }\right)-V\left(a_{t+i-1},o_{t+i};\theta_{a}^{\prime },\theta_{q}^{\prime }\right)\right]\log\pi \left(a|a_{t+i-1},o_{t+i};\theta_{a}^{\prime }\right) \end{array}$$

(5) $$\begin{array}{c} -\lambda \sum_{a\in A}\pi \left(a|a_{t+i-1},o_{t+i};\theta_{a}^{\prime }\right)\log\pi \left(a|a_{t+i-1},o_{t+i};\theta_{a}^{\prime }\right)\}. \end{array}$$

The hyperparameter λ is the weight of the entropy term.

**Figure 4:**
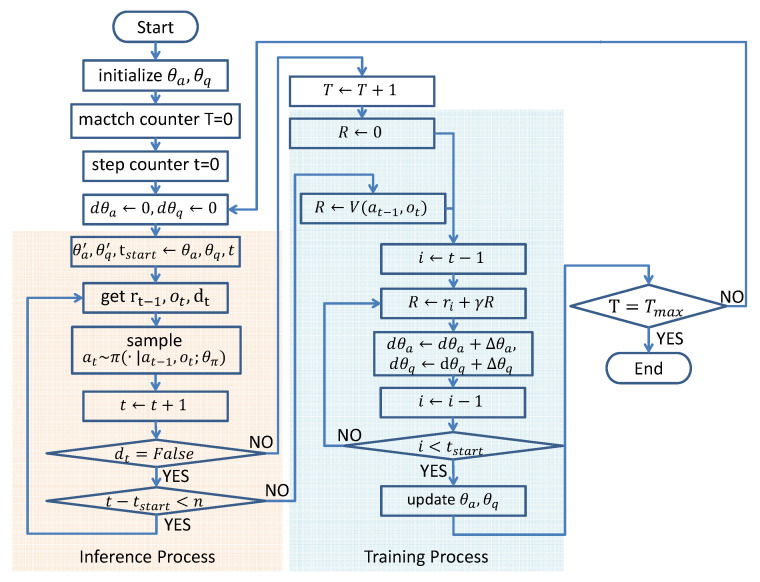
The algorithm flow chart of MA3C. The inference and training processes are executed in turn until the total number of matches, *T*, reaches $T_{max}$.

Algorithm 1 shows the complete algorithm. Additionally, Figure 4 shows the algorithm flow chart.
**Algorithm 1. **Mean Asynchronous Advantage Actor-Critic (pseudo code for each thread)1:Assume global shared counter T=02:Randomly initialize global shared parameter vectors $\theta_{a}$, $\theta_{q}$ and thread-specific parameter vectors $\theta_{a}^{\prime }$, $\theta_{q}^{\prime }$3:Initialize step counter t←14:Set $a_{0}\leftarrow no\;operation$5:**while**$T<T_{max}$**do**6:    Set the gradients $d\theta_{a}\leftarrow 0$ and $d\theta_{q}\leftarrow 0$7:    Set the parameter $\theta_{a}^{\prime }\leftarrow \theta_{a}$, $\theta_{q}^{\prime }\leftarrow \theta_{v}$ and $t_{start}\leftarrow t$8:    Get observation $o_{t}$9:    **repeat**10:        Sample $a_{t}$ according to policy $\pi (a_{t}|a_{t-1},o_{t};\theta_{a}^{\prime })$11:        Receive reward $r_{t}$ and observation $o_{t+1}$ from MA3C-Bot12:        t←t+113:    **until** terminal $s_{t}$**or** $t-t_{start}==n$14:    **if** terminal $s_{t}$ **then**15:        T←T+116:        R←017:    **else**18:        $R\leftarrow V(a_{t-1},o_{t})$19:    **end if**20:    **for** $i\in \{t-1,\cdots ,t_{start}\}$ **do**21:        $R\leftarrow r_{i}+\gamma R$22:         //Compute the gradient of the n-step Q-loss23:        $d\theta_{q}\leftarrow d\theta_{q}+{▿}_{\theta_{q}^{\prime }}{\left(R-Q\left(a_{i-1},o_{i},a_{i};\theta_{q}^{\prime }\right)\right)}^{2}$24:         //Compute the policy gradient using Equation (5)25:        $d\theta_{a}\leftarrow d\theta_{a}-\Delta \theta_{a}^{\prime }$26:    **end for**27:    Update $\theta_{a}$, $\theta_{a}^{\prime }$ with $d\theta_{a}$ and update $\theta_{q}$, $\theta_{q}^{\prime }$ with $d\theta_{q}$28:**end while**

## 5. Experiments

### 5.1. Experimental Settings

The action space contains three actions, producing three different army units: Zergling, Hydralisk, and Mutalisk. Every 24 frames, MA3C-Bot requests a decision after it finishes a fixed opening build queue, ‘10HatchMuta’. Additionally, the actions are repeatedly executed in the 24 frames if the resources are sufficient.

We select Steamhammer-1.2.3 (http://satirist.org/ai/starcraft/steamhammer/1.2.3/, accessed 10 April 2021), an open-source StarCraft bot, as the opponent bot. It can play all three races in StarCraft and have dozens of opening build orders. As a full-featured starter bot, many bots descended from it. In order to facilitate the analysis, the opening build order of Steamhammer is fixed as the one of ‘11Rax’, ‘9HatchMain9Pool9Gas’, and ‘9PoolSpeed’. The first opening build order corresponds to the race of Terran, and the latter two correspond to the race of Zerg. In order to facilitate the description following, we name Steamhammer with the three opening strategies as ‘Steamhammer-a’, ‘Steamhammer-b’, and ‘Steamhammer-c’, respectively. For each opening build order of Steamhammer, MA3C-Bot plays 1000 matches against it in a map named ‘Benzene’. The RL model is trained during this process.

We select the random policy, randomly selecting an action from the action space, as the baseline. Besides, in order to evaluate the performance of MA3C, we make a comparison with Overkill (https://github.com/sijiaxu/Overkill/tree/v1.2, accessed 10 April 2021), which is designed to solve the macromanagement problem by the traditional Q-learning method. In order to compare our method fairly with it and evaluate its effectiveness, Overkill has the same opening build queue and action space with MA3C-Bot.

We initialize the weights of our RL network with standard normal distribution and utilize the Adam optimizer [35] to train it with the learning rate of 0.0001. The discount factor γ is set as 0.99 to increase the weight of future rewards. The number of parallel actor-learners in this experiment is 4.

### 5.2. Analysis on Learned Strategies

In this section, we visualize the macromanagement that is learned by MA3C through exhibiting the trend of the values $V_{t}$ and the policies $\pi_{t}$ varying with time *t*. Subsequently, we analyze the learned macromanagement through the visualized results and the snapshots of games.

We train the MA3C agent playing against Steamhammer-a with 1000 matches and then test the agent 100 matches against Steamhammer-a. The outputs of the LSTM layer at each time are recorded as the state feature $s_{t}$. Subsequently, we use t-SNE [36] to project the state feature $s_{t}$ on a 2D space and color these projected points according to the time *t*, the policy $\pi_{t}$, and the value $V_{t}$ evaluated by MA3C, respectively. $\pi_{t}$ is the output of the policy network at the time *t* and it means the probabilities of selecting Zergling, Hydralisk, or Mutalisk to produce.

**Figure 5:**
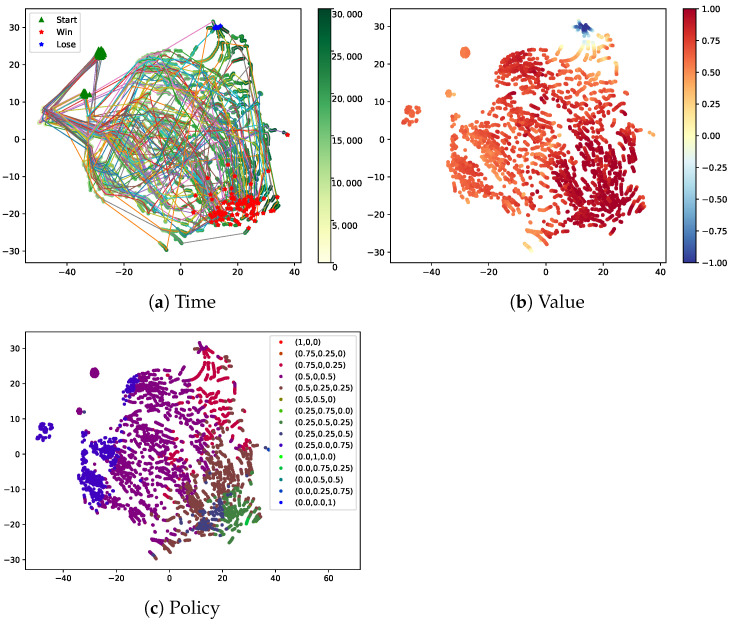
The t-SNE figures of the states that are generated by the learned policy in 100 matches. The opponent bot, Steamhammer, uses the opening strategy of 11Rax. The states are colored based on time, value, and policy, respectively (Best viewed with zoom). (**a**): The states of the beginning, the winning end, and the losing end are marked as green triangles, red asterisks, and blue asterisks, respectively. Other states are masked as green points, and they are colored based on the times according to them. The points in the same match are connected with thin lines. (**b**): The states are colored based on the value for them evaluated by the learned value network. The value estimations roughly fit the real outcomes of the matches. (**c**): The states are colored based on the action selection probability for them computed by the learned policy network. These probabilities are divided into 15 classes depending on which basic probability vector is the closest. The 15 basic probability vectors are shown in the upper right. In the most cases, the trained agent tends to mainly produce Mutalisk in the early stage, produce Mutalisk and Zergling equitably in the middle stage, and produce each unit equitably in the final stage.

In order to facilitate the visualization, we mark the beginning states, the winning end states and losing end states as green triangles, red asterisks, and blue asterisks, respectively, and connect the points of the same match with thin lines in Figure 5a.

For clarity, we divide all of the available policies into 15 classes and color points based on the class. We select 15 basic policy vectors, $b_{1}={\left(1,0,0\right)}^{T},b_{2}={\left(0.75,0.25,0\right)}^{T},\cdots ,b_{15}={\left(0,0,1\right)}^{T}$, and assign the policy $\pi_{t}$ to the according class based on the closest basic vector from it.

Next, we analyze the learned policies by combining the t-SNE graphics and the snapshots of games or the charts of the main army over time. While explaining the policies, we will point out the special benefit brought by MA3C.

From Figure 5a, we can observe that the time of states is increasing from left to right. In the early stage (from 0-th to 12,000-th frame), MA3C-Bot implements the total actions in the opening strategy and then executes a few decisions made by the RL agent. The trajectories in this stage are very dense. In the medium stage (from 12,000-th to 20,000-th frame), the trajectories are loose and chaotic for the randomness of policies and the uncertainty of state transition. In the final stage (from 20,000-th to 30,000-th frame), the trajectories gather in several areas. Combined with Figure 5b, the color of these points becomes deeper over time and the value estimations of states become more accurate. Through Figure 5c, we observe that the policy $\pi_{t}$ is changed from ${\left(0.25,0.0,0.75\right)}^{T}$ to ${\left(0.5,0.0,0.5\right)}^{T}$ and to the mixtures of ${\left(0.5,0.25,0.25\right)}^{T}$, ${\left(0.25,0.5,0.25\right)}^{T}$, and ${\left(0.25,0.25,0.5\right)}^{T}$. In other words, the trained agent tends to mainly produce Mutalisk in the early stage, produce Mutalisk and Zergling equitably in the middle stage, and produce each unit equitably in the final stage.

**Figure 6:**
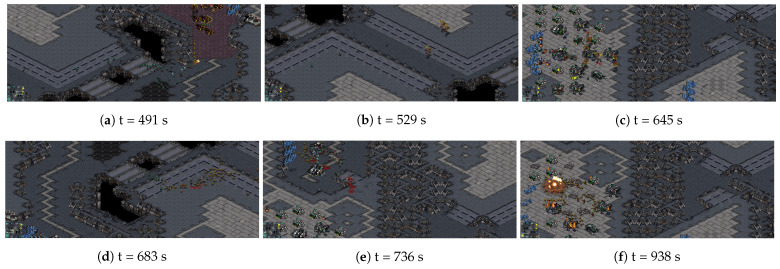
Some snapshots of one match at different times (Best viewed with zoom). (**a**): A few Zerglings of our side and the Marines of the opponent’s side are locked in a face-off. (**b**): The Mutalisks of us attack the opponent’s relief troops. (**c**): The number of our army increases sharply and the Mutalisks’ target is changed to the opponent’s bases. (**d**): The Marines pull back to defeat the opponent’s bases, and they are pursued by massive Zerglings. (**e**): Few opponent’s armies are survived. (**f**): Our army destroys the total opponent’s buildings.

Combining with some snapshots of one match that is shown in Figure 6, we have a better understanding of those policies mentioned in the previous paragraph. In the early stage, a few Zerglings of ours and the marines of opponent’s are locked in a face-off (Figure 6a), while the Mutalisks attack the opponent’s relief troops (Figure 6b). For the help of defense construction, the defensive battle is not difficult. Thus, most of the resources are used to produce Mutalisks rather than Zerglings. In the medium stage, the number of our army increases sharply and the Mutalisks’ target is changed to the opponent’s bases (Figure 6c). The marines pull back to defeat the bases, but they are pursued by massive Zerglings (Figure 6d). In order to effectively eliminate the marines, more Zerglings should be produced. In the final stage, there are few opponent’s army left (Figure 6e), and our army destroys the opponent’s buildings completely (Figure 6f). Because a few in the opponent’s army are left, any kind of army unit can be produced to destroy the opponent’s buildings. In a word, the learned macromanagement can adjust the proportion of different units to cope with the demands of tactic and resource management modules. Thus, the victory is attributed to not only the MA3C’s decision-making, but also its outstanding coordination with tactic and micromanagement modules.

Specially, there are some red points (the label of them is ${\left(0.75,0.0,0.25\right)}^{T}$) in the right upper corner of Figure 5c, which means that the agent would change its policy in some particular situations. When combining with Figure 5a, most of the trajectories according to these red points are ended with winning. That is to say, the agent averts failure to some extent through switching the policy to ${\left(0.75,0.0,0.25\right)}^{T}$.

In a word, the learned macromanagement is not only adapted to the game rules and the policy of the opponent bot, but it also cooperates well with the other modules of MA3C-Bot.

### 5.3. Results Comparison

**Figure 7:**
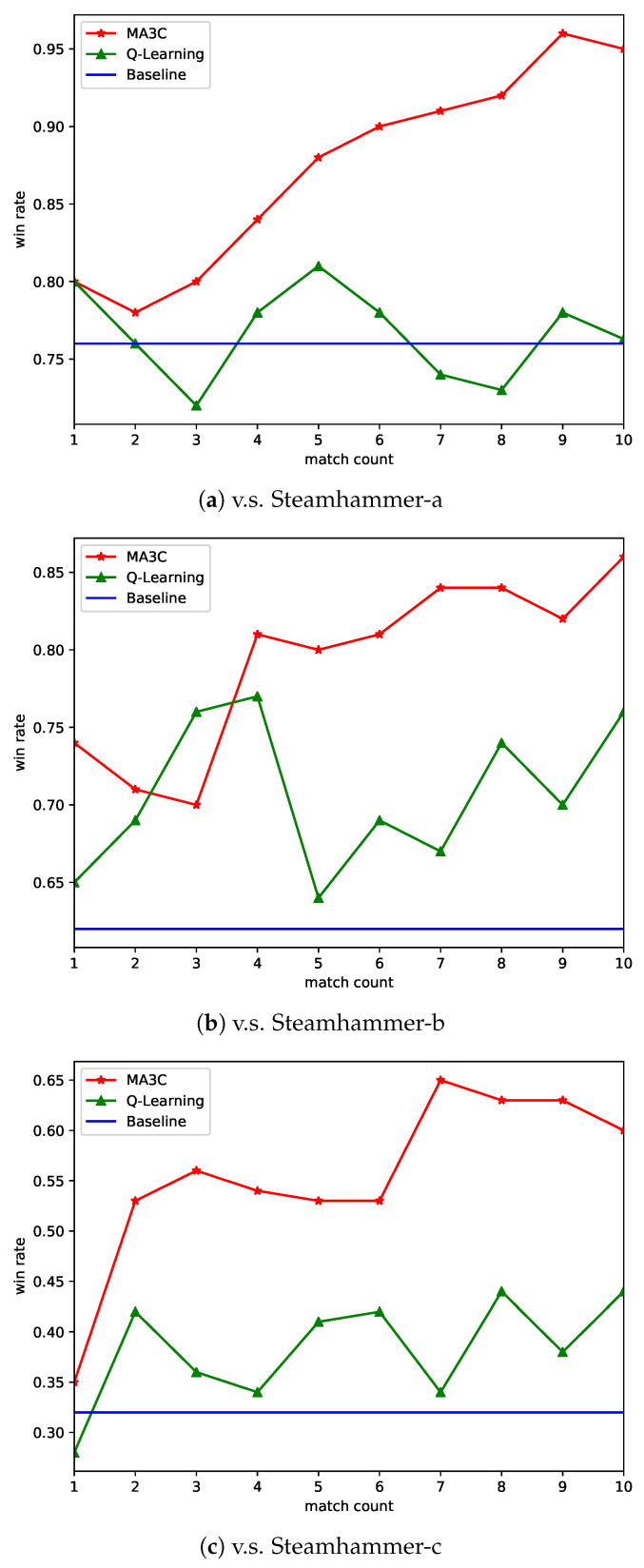
The win rate of each 100 matches over time in the training process. Comparing with Q-learning method, MA3C significantly improves the performance. Against the weaker two opponents, MA3C achieves very high rate of winning, about 90%. For the hardest case, the learned policy still improves win rate about 30%.

Figure 7 shows the win rates in the training processes of MA3C-bot playing against Steamhammer-a,b,c. The baseline is the win rate of Overkill with random policy playing 100 matches against Steamhammer. The baseline in three different cases are decreased from 76% to 62%, and to 32%, which means that Steamhammer-a is the weakest opponent, followed by Steamhammer-b, and the strongest is Steamhammer-c.

We record the average win rate of each 100 matches in the training procedure of MA3C and the Q-learning method implemented in Overkill. The results show that the performance of the Q-learning method is very unstable. While the win rate of our method steadily increases and it shows a tendency toward stabilization, excluding the instability of early stage. When comparing with the baseline and the Q-learning method, our method significantly improves the performance. Against the weaker two opponents, our agent achieves a very high rate of winning, approximately 90%. For the hardest case, the learned policy still improves a win rate of about 30%.

### 5.4. The Generalization Ability of Our Method

To evaluate the generalization ability of our method, we select a RL agent that is trained against Steanhammer-b and test it against six different Steamhammer bots. These Steamhammer bots is the same as Steanhammer-b, but the opening strategies of them are replaced with 9PoolSpeed, OverhatchLing, OverhatchMuta, OverpoolSpeedling, Overgas11Pool, and Overpool9Gas, respectively. The RL agent plays 100 matches against each Steamhammer bot, and the win rates against different bots are shown in Table 3. We also test the random policy against these bots as the baseline.

When comparing with the baseline, the trained agent significantly improves the win rate. This result shows that the learned macromanagement can make reasonable decision for the situations that are unseen in the training process and can adapt the change of the opponent policy to a certain extent.

## 6. Discussion

Generally, the experimental results demonstrate that MA3C can efficiently solve the POMDP and the uncertainty problem in StarCraft, and it has high sample efficiency. The policy learned by MA3C from playing against the strong opponent can deal with the unseen opponents’ policy well to a certain extent.

The benefit from the calculation method of the policy gradient, the variance of the gradient is smaller, so the training process is more stable and efficient. The win-rate curves that are shown in Figure 7 can confirm this point. The nearby points correspond to similar values and similar policies, but they do not always correspond to similar times, as shown in Figure 5. This phenomenon shows that the encoder in MA3C can encode the historical observation sequence well and prepare for the subsequent processes of value estimation and behavioral decision-making. The snapshots of a match in Figure 6 can help us to understand the reasons behind the learned policy. It is worth noting that this policy is learned through trial and error, rather than the information of other modules. Thus, when other modules are updated or replaced, it is still possible for MA3C-bot to obtain an appropriate policy through training. Although there is a cycle of restraint between strategies in the game of StarCraft, it seems that the learned policy still has a certain generalization ability, as shown in Table 3. This means that MA3C-bot has learned some general decisions to deal with the states that it has never seen before.

However, the algorithm MA3C also has several disadvantages: firstly, the effectiveness of Equation (5) is related to the accuracy of the Q-value estimation. When the biases of the estimated Q-values and the real values are large, the policy gradient that is computed by MA3C would have greater bias than the one computed by the original A3C. Secondly, the exploration ability of MA3C is limited. If the capacity of the opponent bot is very highly capable, the macromanagement task would be hard and the learned policy would be extremely complex. In this case, the exploration ability provided by entropy regularization is difficult to assist MA3C to learn the appropriate policy.

## 7. Conclusions and Future Work

In this paper, we have proposed a novel reinforcement learning method, Mean Asynchronous Advantage Actor-Critic (MA3C), to learn the macromanagement in StarCraft. MA3C tackles the POMDP problem through using LSTM to encode the history queue, and reduce the variance of the policy gradient through computing the approximate expected gradient. Besides, multiple parallel RL processes efficiently reduce the training time. The experimental results demonstrate that MA3C has the ability to learn the appropriate policies and significantly improves the win rate. The RL agent trained against a stronger bot can play against the unseen bots well, which means that the learned macromanagement can generalize to the unseen situations and adapt the change of the opponent policy in a certain extent. Subsequently, we propose a novel visualization method to interpret the policy learned by MA3C. The analysis suggests that the RL agent’s decision is based on not only the game rules and the state of two sides, but also the capability of the other modules in our bot. Thus, MA3C can be able to efficiently learn the appropriate policies to adapt the other modules in the bot and improve the win rate.

We plan to introduce the ensemble Q-network [23,37,38] or weighted Q estimates [39,40] to reduce the biases of the estimated Q-values and improve the stability of our algorithm. Another important aspect would be improving the exploration ability, which will further enhance the performance of our algorithm in the face of a stronger opponent bot.

## Figures and Tables

**Table 1:** The comparison of the methods to learn macromanagement.

	Category	Training Time	Appropriate for Other Modules	Computing Resources	Parameters Magnitude	Opponent
[13]	SL	Short	Not	Medium	Medium	Built-in Bot
[14,15]	EC	Long	Yes	Large	Small	Built-in Bot
[31,32,33]	SL+RL	Very Long	Yes	Huge	Huge	Professional Player
[16]	RL	Long	Yes	Medium	Medium	Built-in AI
Our Method	RL	Medium	Yes	Medium	Medium	Steamhammer Bot

**Table 2:** The total raw features of observations.

Feature		Type
	Time	continuous
Map	Width	continuous
	Height	continuous
Race	IsEnemyZerg	discrete
	IsEnemyTerran	discrete
	IsEnemyProtoss	discrete
	Unknow	discrete
Economy	Supply	continuous
	SupplyUsed	continuous
	OurAllBattleUnit	continuous
	EnemyAllBattleUnit	continuous
	BuildingScore	continuous
	GatheredMinerals	continuous
	GatheredGas	continuous
	Minerals	continuous
	Gas	continuous
	OurWorkers	continuous
	EnemyWorkers	continuous
	OurBases	continuous
	EnemyBases	continuous
Technology	hasHive	discrete
	hasLair	discrete
	hasHatchery	discrete
	hasPneumatizedCarapace	discrete
	Other Technologies of Us	discrete
Buildings & Army	OurHatchery	continuous
	OurZergling	continuous
	Other Buildings&Units of Us	continuous
	EnemyHatchery	continuous
	EnemyZergling	continuous
	Other Buildings&Units of Enemy	continuous

**Table 3:** The win rates of the trained RL agent against Steamhammer bots using different opening strategies.

**Opening Strategy**	**9PoolSpeed**	**OverhatchLing**	**OverhatchMuta**
Random	0.32	0.62	0.51
MA3C	0.57	0.71	0.74
**Opening Strategy**	**OverpoolSpeedling**	**Overgas11Pool**	**Overpool9Gas**
Random	0.47	0.62	0.83
MA3C	0.73	0.66	0.84

## Data Availability

No new data were created or analyzed in this study. Data sharing is not applicable to this article.

## Abbreviations

The following abbreviations are used in this manuscript:

RL	Reinforcement Learning
MA3C	Mean Asynchronous Advantage Actor-Critic
RTS	Real-Time Strategy
DQN	Deep Q-Network
POMDPs	Partially Observable Markov Decision Processes
DRQN	Deep Recurrent Q-Network
LSTM	Long Short Term Memory
A3C	Asynchronous Advantage Actor-Critic
t-SNE	t-Distributed Stochastic Neighbor Embedding
